# Sleep syncope—A systematic review

**DOI:** 10.3389/fcvm.2022.973368

**Published:** 2022-10-06

**Authors:** Priya L. Raj, Robert S. Sheldon, Diane Lorenzetti, David L. Jardine, Satish R. Raj, Bert Vandenberk

**Affiliations:** ^1^Department of Cardiac Sciences, Libin Cardiovascular Institute, Cumming School of Medicine, University of Calgary, Calgary, AB, Canada; ^2^Department of Community Health Sciences, Cumming School of Medicine, University of Calgary, Calgary, AB, Canada; ^3^Health Sciences Library, University of Calgary, Calgary, AB, Canada; ^4^Department of General Medicine, Christchurch Hospital, Christchurch, New Zealand; ^5^Department of Cardiovascular Sciences, KU Leuven, Leuven, Belgium

**Keywords:** vasovagal syncope, sleep syncope, supine syncope, prodromes, systematic review

## Abstract

**Background:**

Sleep syncope is a subtype of vasovagal syncope in which patients experience syncope after awakening from their sleep. The aim was to investigate the association of clinical characteristics and gastrointestinal symptoms with syncope, as well as the body position in which symptoms began.

**Methods:**

A systematic search of studies was performed in MEDLINE and EMBASE without language restrictions, from inception to 9 January 2022. Studies were included if they reported data on the proportion of patients who experienced symptoms (nausea, vomiting, abdominal pain, and diarrhea) associated with syncope.

**Results:**

Data were included for 116 patients in 13 studies. Patients were 46.9 ± 4.3 years and 61.4% were female. In 52.5% of patients, a supine body position at the time of syncope was reported. A history of phobias was reported by 67.6% of patients, and 96.5% of patients also had typical daytime vasovagal syncope. In the 5 studies reporting the results of head-up tilt testing (*n* = 77), 90.9% of patients had positive tests. Gastrointestinal symptoms were present in the majority of patients with reported rates of 65.6% for upper gastrointestinal symptoms and 86.0% for lower gastrointestinal symptoms.

**Conclusion:**

Patients with sleep syncope patients are predominantly female with a history of daytime vasovagal syncope. Gastrointestinal symptoms are present in the majority of patients and is therefore an important feature of sleep syncope.

## Introduction

Supine vasovagal syncope, also known as sleep syncope, is a subtype of vasovagal syncope in which a patient experiences the onset of recurrent syncope nocturnally, interrupting their sleep (1). While vasovagal syncope usually occurs during an upright position or in the presence of pain, injury, trauma, or medical settings, none of these commonly occur while asleep. In fact, assuming a supine position is a recommended strategy to prevent syncope during a vasovagal response. However, syncope in a supine position is still possible (1). Due to limited awareness of sleep syncope and its infrequency, the diagnosis is often missed (2). Sleep syncope is often misdiagnosed as epilepsy, since seizures are a common reason of transient loss of consciousness in the supine position (3).

The physiologic cascade culminating in sleep syncope is poorly understood. Physiologic stress is possible, but unlikely given that the subject is asleep at the onset of the spells. Many subjects with sleep syncope experience gastrointestinal symptoms such as nausea, abdominal pain, and the urge to defecate. These symptoms often awaken patients, causing them to stand up and head to the bathroom. These initial symptoms may be due to vagal efferent traffic in keeping with the onset of the vasovagal reflex cascade (4), and for most the ensuing upright posture culminates in hypotension and syncope.

To better understand the progression of the symptoms of sleep syncope, we conducted a systematic review regarding the clinical characteristics and symptoms of sleep syncope patients. The focus was on the presentation and time of the onset of symptoms, the association of gastrointestinal symptoms with sleep syncope, and the body position in which syncope occurred.

## Materials and methods

The search strategy for this study was designed based on theoretical deduction, and the methodology adhered to the preferred reporting items for systematic reviews and meta-analyses [PRISMA (5)] statement. The study protocol was not registered or published prior to performing the search strategy. The data underlying this article will be shared on reasonable request to the corresponding author.

### Data sources and search strategy

A systematic literature search was performed in the MEDLINE and EMBASE databases from inception until June 8, 2022. The search combined subject headings (e.g., MEDLINE MeSH) and keywords (title/abstract words and synonyms) from two concepts: (1) vasovagal/supine syncope and (2) sleep. The detailed search string is presented in Supplementary Material 1. There were no language restrictions applied. The reference lists of included studies were also reviewed to identify additional studies of relevance.

### Eligibility criteria

Observational studies (case reports, case series, case-control studies, and cohort studies), and both randomized and non-randomized clinical trials on patients with sleep syncope were eligible for inclusion. Inclusion criteria were limited to studies published in any language that reported the symptoms of patients with sleep syncope as proportions or odds ratios. The diagnosis of sleep syncope was made according to the local institution but was verified during this review process. No restrictions were made with regards to patient characteristics or frequency of syncope episodes.

### Study screening

**FIGURE 1:**
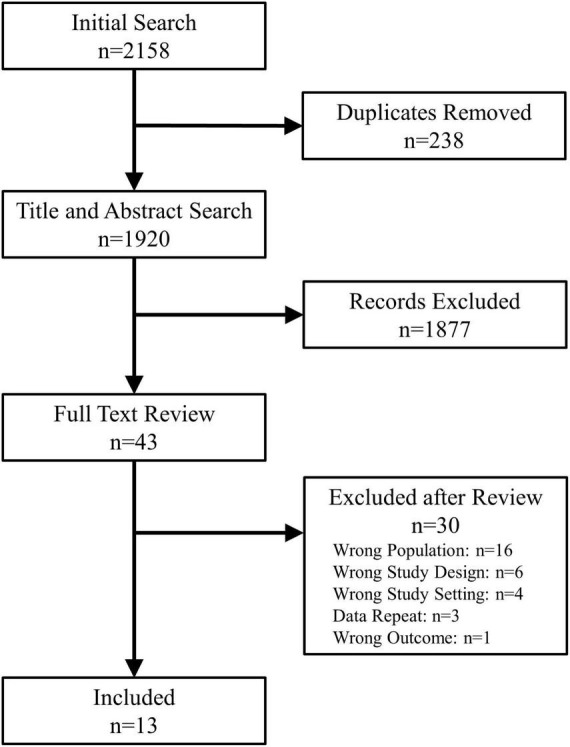
PRISMA flowchart.

Titles and abstracts were screened in duplicate (P.L.R. and B.V.) for relevance. The remaining full-text manuscripts were reviewed in duplicate (P.L.R. and B.V.) to verify the study design, patient population and outcome. Manuscripts published by the same research groups were reviewed in detail to assess whether they included unique patients. In case of duplicate patients, studies by the same group could only be included if they presented unique data, not present in other manuscripts. Discrepancies were resolved through consensus opinions and if required by discussion with the senior authors (R.S.S. and S.R.R.). The study selection process is shown in Figure 1.

### Data extraction

Data were extracted in duplicate by two of the reviewers (P.L.R. and B.V.) and included patient demographics, study design, number of syncopal events, body position at syncope, and frequency of specific gastrointestinal symptoms. Any discrepancies regarding data extraction were resolved in consultation with senior authors on the team (R.S.S. and S.R.R.). As study data were not reported homogeneously or completely across studies not all data fields were complete.

The symptoms commonly associated with sleep syncope that were recorded included nausea, vomiting, abdominal pain, and diarrhea. The four symptoms were compiled in two categories: upper gastrointestinal symptoms (nausea and vomiting) and lower gastrointestinal symptoms (abdominal pain and diarrhea). Other symptoms that were collected included: episodes during childhood, profuse sweating or diaphoresis, significant bradycardia (<30 bpm) or asystole (>3 s) during monitored episodes, and vagotonia. The latter was defined as several vasovagal episodes, usually associated with severe bradycardia or asystole over a few hours (6).

### Quality assessment

The risk of bias was assessed independently and in duplicate by B.V. and R.S.S. and discrepancies were resolved by consensus. Cohort studies and case-control studies were evaluated using the Newcastle-Ottawa scale (NOS) (7). The originally defined measures were slightly modified to be used in a syncope cohort. The measures used were:

i Selection:
  a. Adequate case definition (1 point)
  b. Representativeness of the cases (1 point)
  c. Selection of controls (1 point)
  d. Definition of controls (1 point)
ii Comparability:
  a. Comparability of cases and controls: (2 points)
iii Exposure and outcome:
  a. Ascertainment of syncope (1 point)
  b. Same ascertainment for controls (1 point)
  c. Adequacy of follow-up (1 point)

A maximum of 9 points was awarded. A score ≤3 was considered poor quality, between 4 and 6 as fair quality, and ≥7 as good quality. Case reports and case series were evaluated using a previously published tool by Murad et al. (8). Causality statements regarding the use of drugs were excluded. A maximum of 6 points was awarded: 1 point for selection criteria, 2 points for ascertainment, 2 points for causality and 1 point for reporting. A score ≤2 was considered poor quality, between 3 and 4 as fair quality, and ≥5 as good quality. For visualization, scores received color coding with green, orange, or red for good, fair, and poor quality, respectively.

### Statistical analysis

Continuous data are presented as mean ± standard deviation (SD) and categorical variables as absolute number and percentages (%). If applicable, data were calculated or transformed to present data in the most uniform way. Weighted means and standard deviations were calculated where applicable. Analysis was performed using Stata 17.0 (Stata Corp. LLC, TX, USA).

## Results

### Study selection

Among the 1,869 unique studies identified by the search strategy, 43 studies were retrieved for full-text review (Figure 1). Of these, 13 met the inclusion criteria and were included in this review (Table 1) (6, 9–20). The studies by Busweiler et al. (10) and Jardine et al. (6) reported on patients from the same cohort; however, the study by Busweiler et al. (10) reported unique data on the body position and upper gastrointestinal symptoms which was not available in the larger, more recent cohort presented in Jardine et al. (6). As such, the study by Busweiler et al. (10) was included, but only for the specific research questions. The control data from 3 papers were excluded as the data were not obtained from sleep syncope patients (6, 10, 14). Furthermore, one of two case studies from a fourth paper was excluded because the subject did not have sleep syncope (16). Of the included studies, 5 were case reports, 3 case series, 3 cohort studies, and 2 case-control studies.

**TABLE 1:** Overview of final study selection.

First author (year)	Design	Size, *n*	Age, years	Female, *n* (%)	Observation duration, months
Atsuumi et al. (9)	Case report	1	53.0	0 (0.0%)	24
°Busweiler et al. (10)	Retrospective case-control	54	46.0 ± 2.1	35 (64.8%)	96
Eguchi et al. (11)	Case report	1	66.0	0 (0.0%)	12
Iskos et al. (12)	Retrospective case series	2	28.5 ± 0.5	0 (0.0%)	11 ± 3
°Jardine et al. (6)	Prospective cohort	69	47.0 ± 15.0	46 (66.7%)	180
*Kapoor (13)	Prospective cohort	9			
*Khadilkar et al. (14)	Retrospective cohort	5			
Marrison and Parry (15)	Case report	1	65.0	0 (0.0%)	
Overdijk et al. (16)	Case report	1	45.0	1 (100%)	
Rytlewski et al. (17)	Case report	1	41.0	1 (100%)	60
Shahab et al. (18)	Retrospective case series	5	44.4 ± 13.8	2 (40.0%)	
Tewfik et al. (19)	Retrospective case-control	19			70
Xu et al. (20)	Retrospective case series	2	50.5 ± 24.5	1 (50.0%)	36 ± 24

*Subgroup from larger cohort. °Includes data from the same cohort. Only unique data presented in the study by Busweiler et al. was used in analysis of clinical characteristics and symptoms. Continuous variables are presented as mean ± standard deviation if available.

### Study quality

The risk of bias assessment for each study is shown in Table 2. The case reports and case series had a mean score of 4.4 (maximum 6, median 5), ranging 3–6. Three studies were deemed of fair quality (15, 16, 18) and 5 of good quality (9, 11, 12, 17, 20). The modified NOS scores had a mean score of 6.6 (maximum 9, median 7), ranging 5–8. Two studies were deemed of fair quality (13, 14), and 3 of good quality (6, 10, 19).

**TABLE 2:** Quality assessment of included studies.

A. Case reports and case series					

First author (year)	Selection	Ascertainment	Causality	Reporting	Risk of bias

Max score	1	2	2	1	6
Atsuumi et al. (9)	1	2	2	1	6
Eguchi et al. (11)	0	2	2	1	5
Iskos et al. (12)	0	2	2	1	5
Marrison and Parry (15)	0	1	1	1	3
Overdijk et al. (16)	0	1	1	1	3
Rytlewski et al. (17)	0	2	2	1	5
Shahab et al. (18)	0	1	1	1	3
Xu et al. (20)	0	2	2	1	5

**B. Cohort studies and case-control studies**					

**First author (year)**	**Selection**	**Comparability**		**Exposure/outcome**	**Risk of bias**

**Max score**	**4**	**2**		**3**	**9**

Busweiler et al. (10)	4	2		2	8
Jardine et al. (6)	4	2		2	8
Kapoor (13)	2	0		3	5
Khadilkar et al. (14)	3	1		1	5
Tewfik et al. (19)	4	1		2	7

### Patient characteristics

**FIGURE 2:**
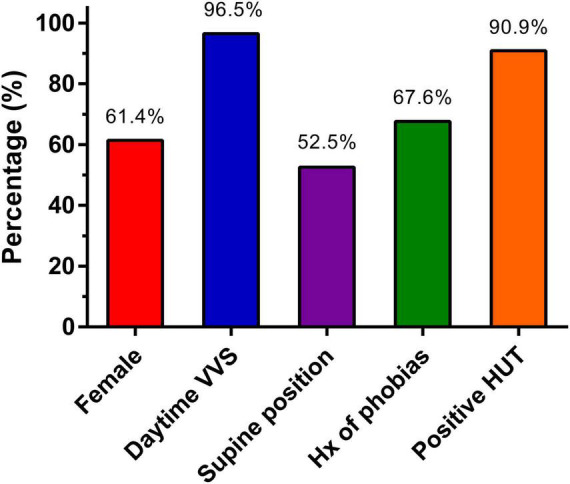
Overview of patient characteristics.

Among all included studies there were 116 unique patients diagnosed with sleep syncope. The patients had a mean age of 46.9 ± 4.3 years and 61.4% were female. A detailed history of the patients is presented in Figure 2 and Table 3. All the included patients had a history of prior syncope and 96.5% also had daytime vasovagal syncope. Syncope during childhood were reported in 44 of 81 patients (54.3%). A supine position at the time of syncope was reported in 52.5% patients (53 of 101 available), while in the remaining the syncope occurred shortly after standing. In 2 available studies, 50 of 74 patients (67.6%) had a history of phobias. Results of head-up tilt testing was reported in five studies (*n* = 77), and they were positive in 70 patients (90.9%). No detailed description of phobias or Holter findings were available.

**TABLE 3:** Study participants syncope history.

First author (year)	Syncope history, years	Sleep syncope prevalence^#^	Supine, *n* (%)	Daytime VVS, *n* (%)	Childhood syncope, *n* (%)	Phobias, *n* (%)	Tilt positive, *n* (%)
Atsuumi et al. (9)	1	10	1 (100%)		0 (0.0%)		
°Busweiler et al. (10)	Lifetime	5.4 ± 0.8	18 (32.7%)	54 (100%)	27 (50.0%)	40 (74.5%)	47 (87.0%)
Eguchi et al. (11)	1	4	1 (100%)		0 (0.0%)		
Iskos et al. (12)	0.9 ± 0.3		2 (100%)				
°Jardine et al. (6)	6.2 ± 7	2.0 ± 3 per year		68 (98.6%)	41 (58.0%)	49 (71.0%)	63 (91.3%)
*Kapoor (13)			9 (100%)	9 (100%)			
*Khadilkar et al. (14)			5 (100%)			1 (20%)	
Marrison and Parry (15)	21	3	1 (100%)		0 (0.0%)		1 (100.0%)
Overdijk et al. (16)			1 (100%)		1 (100%)		1 (100.0%)
Rytlewski et al. (17)	20	Every 8 to 12 months	0 (0.0%)	1 (100%)	1 (100%)		0 (0.0%)
Shahab et al. (18)			5 (100%)	4 (80.0%)	1 (20.0%)		5 (100.0%)
Tewfik et al. (19)	0.5	3	8 (42.1%)				
Xu et al. (20)	6.0 ± 1.0		2 (100%)	1 (50.0%)	0 (0.0%)		

*Subgroup from larger cohort. °Includes data from the same cohort. ^#^Absolute number or frequency as reported in original studies. Only unique data presented in the study by Busweiler et al. was used in analysis of clinical characteristics and symptoms.

### Upper gastrointestinal symptoms

A total of six studies reported the prevalence of upper gastrointestinal symptoms (Table 4). From these reports, 65.6% of patients experienced nausea and 42.9% experienced vomiting. Combined upper gastrointestinal symptoms were present in 65.6% of patients. When evaluating only the 5 studies who specified the presence of symptoms prior to syncope, the prevalence of nausea prior to syncope was 90.0%. Only the study by Jardine et al. (6) reported a detailed medical history. There was a trend toward a higher prevalence of gastrointestinal medical conditions in sleep syncope patients (11.6% vs. 3.4%, *p* = 0.06), but the use of proton pump inhibitors and anti-emetics were not significantly different.

**TABLE 4:** Syncope-related symptoms in study participants.

First author (year)	Upper gastrointestinal symptoms			Lower gastrointestinal symptoms			Other	
			
	Nausea, *n* (%)	Vomiting, *n* (%)	Composite, *n* (%)	Defecation urge/abdominal pain, *n* (%)	Diarrhea, *n* (%)	Composite, *n* (%)	Diaphoresis, *n* (%)	Bradycardia/asystole, *n* (%)
Atsuumi et al. (9)								1 (100%)
°Busweiler et al. (10)	33 (61.1%)		33 (61.1%)	39 (72.2%)		39 (72.2%)	35 (64.8%)	
Eguchi et al. (11)				1 (100%)		1 (100%)	1 (100%)	
Iskos et al. (12)								2 (100%)
°Jardine et al. (6)				57 (82.6%)		57 (82.6%)		
*Kapoor (13)				9 (100%)		9 (100%)		
*Khadilkar et al. (14)								
Marrison and Parry (15)	1 (100%)	1 (100%)	1 (100%)				1 (100%)	
Overdijk et al. (16)	1 (100%)		1 (100%)	1 (100%)	1 (100%)	1 (100%)	1 (100%)	1 (100%)
Rytlewski et al. (17)	1 (100%)	1 (100%)	1 (100%)	1 (100%)	1 (100%)	1 (100%)	1 (100%)	1 (100%)
Shahab et al. (18)	5 (100%)	1 (20.0%)	5 (100%)	5 (100%)		5 (100%)	3 (60.0%)	0 (0.0%)
Tewfik et al. (19)								
Xu et al. (20)	1 (50.0%)		1 (50.0%)				1 (50.0%)	2 (100%)

*Subgroup from larger cohort. °Includes data from the same cohort. Only unique data presented in the study by Busweiler et al. was used in analysis of clinical characteristics and symptoms.

### Lower gastrointestinal symptoms

Lower gastrointestinal symptoms were reported in six studies, 86.0% of patients experienced an urge to defecate or abdominal pain (Table 4). Diarrhea was only reported in 2 case reports. Combined lower gastrointestinal symptoms were present in 86.0% of patients. In the 4 studies that specified the urge to defecate or abdominal pain occurring before syncope, all 8 patients (100%) reported this symptom.

### Other symptoms

Profuse sweating or diaphoresis was reported in 43 of 67 patients (64.2%) and significant bradycardia or asystole during witnessed or monitored episodes were present in 7 of 13 patients (53.8%). Only the study by Jardine et al. (6) reported vagotonia, defined as clustered vasovagal episodes over a few hours, which was present in 21 of their patients (30.4%).

## Discussion

This systematic review included 116 unique sleep syncope patients and reports 3 important findings. First, the population was predominantly female, 96.5% of patients also had typical daytime vasovagal syncope, and 54.3% of patients had syncope episodes during childhood. Second, 52.5% of patients were supine at syncope onset, while the remaining lost consciousness shortly after a change to upright body position. Third, the prevalence of any gastrointestinal symptom associated with syncope was 65.6% for upper and 86.0% for lower gastrointestinal symptoms.

Although sleep syncope is considered a rare subtype of vasovagal syncope, the reported prevalence in retrospective studies ranged between 2.5 and 21.3% (6, 14, 19) of patients with vasovagal syncope. This large variability might be related to the difficult differentiation of sleep syncope from situational syncope, for which a detailed history and physical examination is required. Tewfik et al. (19) reported a prevalence of 21.3%, however they could only determine the presence of sleep syncope in 66.9% of patients, whereas Khadilkar et al. (14), and Jardine et al. (6) reported the prevalence as the proportion of patients compared to all who were referred for vasovagal syncope. In the prospective cohort study by Jardine et al. (6) sleep syncope was diagnosed in 2.5% of all referrals to the syncope clinic over a 14-year period. Given the few studies and the low level of evidence, sleep syncope can be considered an underreported clinical entity likely due to the limited awareness amongst physicians.

**FIGURE 3:**
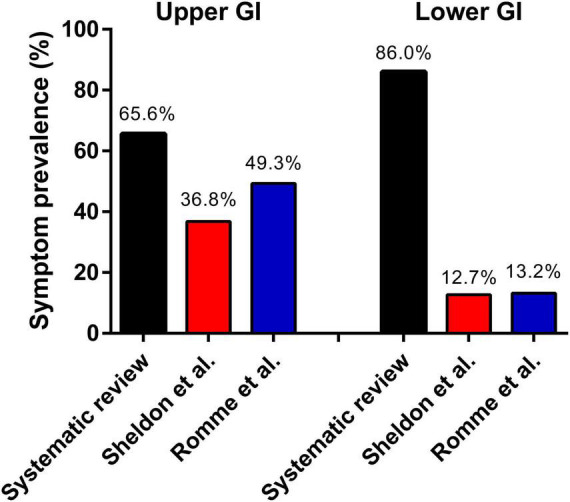
Comparison of gastrointestinal symptoms in literature.

Sleep syncope was initially defined in 2006 by Jardine et al. (1) as loss of consciousness in a non-intoxicated adult occurring during the normal hours of sleep, where the patient wakes up, often with gastrointestinal symptoms, and briefly loses consciousness supine in bed or immediately upon standing. A remarkable high proportion of patients reported phobia-related and childhood syncope. This association has been reported before in family correspondence studies (21, 22). In this current systematic review gastrointestinal symptoms were present in about 86.0% of patients, and in the studies which reported whether gastrointestinal symptoms were present before fainting the rate reached 90%. These rates are significantly higher than those reported in general vasovagal syncope populations (Figure 3), particularly with regards to lower abdominal discomfort. Sheldon et al. (23) described historical criteria to diagnose vasovagal syncope in a comparable population of 235 patients with 61% females and a mean age of 42 years. Nausea or vomiting prior to syncope was present in 36.8% of patients. Romme et al. (24) reported a higher prevalence of 49.3% for nausea during the prodrome in 303 patients included in the FAST study. Abdominal discomfort was reported in 12.7% by Sheldon et al. (23) and syncope or presyncope occurred on the way to the toilet in 13.0%. In Romme et al. (24) the prevalence of abdominal discomfort was 13.2%.

The high prevalence of gastrointestinal symptoms suggests an important pathophysiological role for excessive vagal tone in sleep syncope. Although vasovagal syncope usually occurs in an upright position (23), vasovagal responses or syncope may also occur in a supine position, as is seen with phobia- or emotional-triggered episodes. An upright position should be considered a mediator that increases the likelihood of fainting because of a reduced venous blood return due to blood pooling below the diaphragm. This may explain our finding that about half of patients only faint after standing up to go to the toilet, after not fainting in the preceding supine position. The proportion of patients that were supine at syncope onset, 52.5%, is remarkably high. The accuracy of position ascertainment in the included studies is uncertain, and the rates in different body positions may be influenced by publication bias. A few possible mechanisms predisposing patients to sleep syncope have been suggested (1). During deeper phases of non-rapid eye movement (REM) sleep there is an increased vagal activity, while muscle sympathetic nerve activity is reduced. Also, during sleep baroreflexes may be modified and there is a more inter-dependent relation between respiration and cardiac output. Patients with sleep syncope have similar responses to sympathovagal function tests, but decreased responses to non-baroreflex-mediated function tests (25).

The question remains: what initiates the pathophysiological cascade of sleep syncope? Sleep syncope might be a primarily brain-initiated vasovagal cascade. Although a clear trigger was suggested and treated in a few case reports (11, 15), in general it is believed that the lower and upper gastro-intestinal prodromes are a result of exaggerated vagal activity mediated by the dorsal motor nuclei of the vagus nerve (1, 26). The latter contains parasympathetic motor neurons regulating subdiaphragmatic organs involved with feeding and digestion (26). The activity of the dorsal motor nucleus of the vagus nerve (1, 26) is controlled by local circuits and by inputs from other brain regions including the hypothalamus.

The hypothalamus is considered the primary control center of homeostasis and it acts via 2 effector systems: the autonomic nervous system and the endocrine system (27). The hypothalamus communicates directly with extra-hypothalamic nuclei such as the dorsal motor nucleus of the vagus nerve and the nucleus ambiguous. The nucleus ambiguous contains preganglionic parasympathetic neurons innervating the postganglionic parasympathetic neurons in the heart and is responsible for the cardioinhibitory response. Further, the hypothalamus is closely involved with the neural and hormonal regulation of sleep (28). We therefore hypothesize that there might be a crucial role for the hypothalamus in the initiation of sleep syncope events, potentially triggered by transitions in the sleep/wake cycle or intense dreams such as nightmares. This ties together the gastrointestinal symptoms mediated by the dorsal motor nerve, and the vasovagal cascade mediated by the nearby nucleus ambiguous.

The broad differential diagnosis of sleep syncope can be narrowed with a detailed clinical history, physical examination, electrocardiogram, and electroencephalogram (1, 29). It has even been suggested that the diagnostic gold standard is a detailed history of an eyewitness describing typical features (1). Recognizing vasovagal prodromes can increase the specificity to differentiate vasovagal syncope from other causes of syncope (23). It should be kept in mind that prodromal symptoms are age- and sex-related with decreasing rates with increasing age and lower rates in man versus women (24). However, there was no difference in prodromal symptoms between different body positions (14). Besides typical vasovagal prodrome symptoms, particular attention should be paid to the association of fainting with a history of daytime vasovagal syncope, episodes during childhood, body position, clustering of events within a few hours, and intense triggers, such as pain, smell, and phobias (10, 14). Many of the included studies were missing a detailed description of heart rate, Holter recordings, carotid sinus hypersensitivity, and head-up tilt testing, so the diagnostic potential of these tests is unclear.

In Jardine et al. (6) heart rate response during head-up tilt testing was not different from patients with classic vasovagal syncope. Further, Jardine et al. (6) showed that patients with sleep syncope had a higher prevalence of gastrointestinal disease in the medical history. Fortunately, in their prospective cohort study with a median follow-up of 15 year, the clinical outcome was not significantly different from patients with vasovagal syncope (6). After assessment in their syncope clinic, 24.2% of patients had recurrence of sleep syncope but only 8.1% continued to have a high burden of sleep syncope (6). Only 2 patients (2.9%) of their sleep syncope patients underwent permanent pacemaker insertion during follow-up (6). This illustrates that even in these more symptomatic patients, the insertion of a pacemaker is not required and remains an individual treatment decision.

Future research should focus on a more detailed phenotyping of these patients. This will allow for a more detailed understanding and determine whether this disorder warrants a new diagnostic category as sleep or nocturnal syncope, or merely represents an extremely symptomatic subgroup of classic vasovagal syncope.

### Limitations

The studies included in this systematic review had an overall low level of evidence and limited sample size, as eight studies were either case reports or case series. This reduced the power of this systematic review and prevented us from performing a meta-analysis, but equally illustrates what might be an underreported prevalence of sleep syncope. Given the sparse amount of available data, particularly the absence of patient-level data, and underreported prevalence, bias was assumed but not formally tested. Lastly, the quality assessment of cohort studies and case-control studies was performed using a modified NOS based on the purposes of this specific study cohort, and hence was not externally validated.

## Conclusion

This systematic review on sleep syncope found a predominantly female population with a history of typical daytime vasovagal syncope. About half of patients had syncopal events in a supine position, whereas the remaining fainted shortly after assuming an upright position. Gastrointestinal prodromes are present in most patients, which suggests that this is an important feature of sleep syncope. While observational data suggest that sleep syncope is not rare, it is likely underreported due to limited awareness amongst physicians.

## Data availability statement

The original contributions presented in this study are included in the article/Supplementary material, further inquiries can be directed to the corresponding author.

## Author contributions

PR: design of the work, data collection, data analysis and interpretation, drafting the article, and final approval of the version to be published. BV and SR: conception and design of the work, data collection, data analysis and interpretation, drafting the article, critical revision of the article, and final approval of the version to be published. DL: design of the work, critical revision of the article, and final approval of the version to be published. DJ and RS: conception and design of the work, critical revision of the article, final approval of the version to be published. All authors contributed to the article and approved the submitted version.
