# Supplementary material for: Sleep syncope—A systematic review
Source: Front Cardiovasc Med. 2022 Oct 6;9:973368. doi: 10.3389/fcvm.2022.973368 (PMC9582595; doi:10.3389/fcvm.2022.973368)
Supplement: Supplementary file 1 [file Data_Sheet_1.pdf]

Supplement 1: Search strategy

A. MEDLINE Search Strategy:

1. recurrent supine syncope.m_titl.

2. “Supine Position”/

3. sleep*/ or sleep*.tw,kf.

4. exp Syncope/ or syncope.tw,kf.

5. exp Supine Position/ or supine.tw,kf.

6. 3 or 5

7. 4 and 6

8. animals/ not humans/

9. 7 not 8

B. EMBASE Search Strategy:

1. *“supine position”/ or supine.tw,kw.

2. sleep/ or sleep.tw,kw.

3. exp faintness/ or faintness.tw,kw.

4. 1 or 2

5. 3 and 4

6. animals/ not human/

9. 5 not 6
